# Comparison of 2016–17 and Previous Epizootics of Highly Pathogenic Avian Influenza H5 Guangdong Lineage in Europe

**DOI:** 10.3201/eid2412.171860

**Published:** 2018-12

**Authors:** Pablo Alarcon, Adam Brouwer, Divya Venkatesh, Daisy Duncan, Chrysostomos I. Dovas, George Georgiades, Isabella Monne, Alice Fusaro, Adam Dan, Krzysztof Śmietanka, Vassilios Ragias, Andrew C. Breed, Taxiarchis Chassalevris, Gabriela Goujgoulova, Charlotte Kristiane Hjulsager, Eoin Ryan, Azucena Sánchez, Eric Niqueux, Niina Tammiranta, Siamak Zohari, David A. Stroud, Vladimir Savić, Nicola S. Lewis, Ian H. Brown

**Affiliations:** Royal Veterinary College, London, UK (P. Alarcon);; Animal and Plant Health Agency, Addlestone, UK (P. Alarcon, A. Brouwer, D. Duncan, A.C. Breed, N.S. Lewis, I.H. Brown);; University of Cambridge, Cambridge, UK (D. Venkatesh);; Aristotle University of Thessaloniki, Thessaloniki, Greece (C.I. Dovas, T. Chassalevris);; Ministry of Rural Development and Food, Thessaloniki (G. Georgiades, V. Ragias);; Istituto Zooprofilattico Sperimentale delle Venezie, Padova, Italy (I. Monne, A. Fusaro);; Veterinary Diagnostic Institute, Budapest, Hungary (A. Dan);; National Veterinary Research Institute, Pulawy, Poland (K. Śmietanka);; Department of Agriculture and Water Resources, Canberra, Australian Capital Territory, Australia (A.C. Breed);; University of Queensland, Brisbane, Queensland, Australia (A.C. Breed);; NDRVMI, Sofia, Bulgaria (G. Goujgoulova);; Technical University of Denmark, Lyngby, Denmark (C.K. Hjulsager);; Central Veterinary Research Laboratory, Celbridge, Ireland (E. Ryan);; Central Veterinary Laboratory, Madrid, Spain (A. Sánchez);; French Agency for Food, Environmental and Occupational Health & Safety, Ploufragan, France (E. Niqueux);; Finnish Food Safety Authority Evira, Helsinki, Finland (N. Tammiranta);; National Veterinary Institute and World Organisation for Animal Health Collaborating Center, Uppsala, Sweden (S. Zohari);; Joint Nature Conservation Committee, Peterborough, UK (D.A. Stroud);; Croatian Veterinary Institute, Zagreb, Croatia (V. Savić)

**Keywords:** highly pathogenic avian influenza, HPAI, H5N8, Europe, epizootic, epidemiology, genetic analyses, Europe, influenza, zoonoses

## Abstract

We analyzed the highly pathogenic avian influenza (HPAI) H5 epizootic of 2016–17 in Europe by epidemiologic and genetic characteristics and compared it with 2 previous epizootics caused by the same H5 Guangdong lineage. The 2016–17 epizootic was the largest in Europe by number of countries and farms affected and greatest diversity of wild birds infected. We observed significant differences among the 3 epizootics regarding region affected, epidemic curve, seasonality, and outbreak duration, making it difficult to predict future HPAI epizootics. However, we know that in 2005–06 and 2016–17 the initial peak of wild bird detections preceded the peak of poultry outbreaks within Europe. Phylogenetic analysis of 2016–17 viruses indicates 2 main pathways into Europe. Our findings highlight the need for global surveillance of viral changes to inform disease preparedness, detection, and control.

Highly pathogenic avian influenza (HPAI) is a zoonotic notifiable disease that can cause high mortality rates in most domestic poultry and in some wild bird species. Since 2003, HPAI H5 viruses have been circulating in poultry in many countries (*1*). Periodically these poultry HPAI viruses have been reintroduced into the wild migratory bird population, representing a key risk pathway for its subsequent global spread (*1*–*3*). However, the effect of HPAI infection in both wild and domestic birds is variable and often strain-specific. Wild birds, particularly of the orders Anseriformes and Charadriiformes, are natural hosts of low pathogenicity avian influenza (*4*).

A passive surveillance system of testing wild birds found dead or sick for avian influenza has been in place in European Union (EU) member states since 2005 (Commission Decision 2005/94/EC, replaced with 2010/367/EU), with the objective of timely detection of HPAI subtype H5N1. Laboratory confirmation of HPAI infection following the development of clinical signs (passive surveillance) is the primary method of poultry surveillance in the EU member states, complemented by a serologic active surveillance program (*5*).

During epidemiologic year 2005–06 (epidemiologic years run from October to September of the next year), HPAI H5N1 clade 2.2 virus of the Guangdong H5 lineage spread to a number of countries in Europe, infecting poultry and wild bird populations (*3*). In 2014–15, another virus of the same lineage, HPAI H5N8 clade 2.3.4.4, was introduced into Europe and associated with variable disease severity, including subclinical infection in wild birds and domestic waterfowl (*6*). This H5N8 virus showed unprecedented intercontinental spread to the United States and Canada and was associated with both wild bird infection and, subsequent to local genetic reassortment, large HPAI H5N2 outbreaks in poultry (*7*). 

In October 2016, a novel HPAI H5 clade 2.3.4.4 virus of the Guangdong lineage was detected in Hungary and was subsequently reported in other countries in Europe, infecting many poultry farms and causing both large-scale and sporadic deaths in wild bird populations. The hemagglutinin (HA) gene of this virus was considered phylogenetically distinct from the previous 2014 clade 2.3.4.4 viruses and was nominally suffixed by A (the 2016 clade) or B (the 2014 clade (*8*) but this subclade definition requires verification by the World Health Organization H5 nomenclature group. We describe the epidemiology and genetic characteristics of the 3 major wild-bird mediated epizootics in Europe associated with the Guangdong HPAI H5 lineage.

## Methods

### Epidemiologic Data and Analyses

We collected data from the 3 major HPAI H5 epizootics in Europe: HPAI H5N1 in epidemiologic year 2005–06 (*2*); HPAI H5N8 in 2014–15; and HPAI H5 in 2016–17. For 2016–17, we collected data through July 31, 2017. We obtained epidemiologic data from the Animal Disease Notification System and the Directorate-General for Health and Food Safety, managed by the European Commission, and from country notifications sent to the EU Reference Laboratory for avian influenza (Animal and Plant Health Agency, Weybridge, UK).

We conducted analyses to describe each epizootic, examined the geographic and temporal spread (epidemic curves), and assessed differences in clinical illness and death rates. For spatial analysis, we grouped countries into 4 regions (North, South-West, South-East, and Central Europe) on the basis of the broad migration patterns of the major migratory water bird species affected by HPAI (Technical Appendix Figure 1) (*9*–*14*). A full description of the methods used is presented in the Technical Appendix.

### Viruses’ Sequence Data and Phylogenetic Analyses

We obtained virus HA gene sequence data from countries’ submissions to the EU Reference Laboratory and from GISAID (http://platform.gisaid.org.) We performed phylogenetic analyses on HA sequence data from each epizootic separately. We used IQ-TREE version 1.5.5 software (*15*) to infer maximum-likelihood trees with approximate likelihood ratio test (1,000 replicates) and bootstrap (100 replicates) support values for branches. We down-sampled each dataset using Cluster Database at High Identity with Tolerance to remove sequences with >99.9% sequence identity (*16*). We performed root-to-tip regression analyses using Tempest version 1.5 on the downsampled datasets (*17*). Then, we inferred Bayesian phylogenetic trees from each downsampled dataset using BEAST version 1.8.4 to determine the mean substitution rate and TMRCA (time to most recent common ancestor) (*18*). We annotated the final trees using FigTree version 1.4.3 (http://tree.bio.ed.ac.uk/software/figtree/). Details of criteria and priors used in the analyses are provided in the Technical Appendix.

## Results

### Epizootic Size 

In 2016–17, a total of 1,108 poultry outbreaks were reported in 21 countries in Europe. Extensive farm-to-farm spread, predominantly in ducks, seemed apparent in France, which had >400 farms affected, and Hungary, with >200 farms infected (*19*). Conversely, in 2005–06, a total of 230 poultry outbreaks occurred in 6 countries, mostly located in Romania (86%) and Hungary (13%). In 2014–15, only 13 poultry outbreaks were reported in 5 countries. The estimated number of poultry culled was 8 times higher in 2016–17 than in 2005–06 (Table 1).

**Table 1:** Highly pathogenic avian influenza outbreaks by country in 3 epizootics in Europe*

Country	**H5N1 2005–06 epizootic**				**H5N8 2014–15 epizootic**					**H5N8 2016–17 epizootic**			
	No. poultry infected	No. wild birds infected	No. poultry culled†		No. poultry infected	No. wild birds infected	No. captive birds infected	No. poultry culled†		No. poultry infected	No. wild birds infected	No. captive birds infected	No. poultry culled†
France	1	21	11,700		–	–	–	–		485	51	3	1,529,361
Hungary	29	12	251,948		1	–	–	22,000		238	86	5	2,678,191
Germany	1	220	14,300		5	2	1	58,964		89	738	15	1,150,631
Bulgaria	–	4	–		–	–	–	–		71	13	2	511,832
Poland	–	29	–		–	–	–	–		65	66	–	1,167,282
Romania	197	17	755,372‡		–	–	–	–		45	93	2	2,222
Czech Republic	–	14	–		–	–	–	–		38	39	–	79,308
Italy	–	19	–		1	–	–	31,985		16	6	–	357,049
Spain	–	1	–		–	–	–	––		10	2	–	28,330
Croatia	§	§	§		–	–	–	–		9	12	–	1,546
United Kingdom	–	1	–		1	–	–	6,178		12	23	–	102,849
Netherlands	–	–	–		5	1	–	245,600		8	48	10	202,004
Slovakia	–	2	–		–	–	–	–		8	58	3	351
Greece	–	25	–		–	–	–	–		5	8	–	28,275
Serbia	§	§	§		–	–	–	–		4	20	–	289
Sweden	1	13	692		–	2	–	–		4	30	2	203,053
Austria	–	46			–	–	–	–		2	55	1	1,258
Ukraine	§	§	§		–	–	–	–		2	3	1	10,288
Bosnia and Herzegovina	§	§	§		–	–	–	–		1	1	1	148
Denmark	1	26	102		–	–	–	–		1	49	1	69
FYROM	§	§	§		–	–	–	–		1	1	–	438
Belgium	–	–	–		–	–	–	–		2	3	13	4,047
Finland	–	–	–		–	–	–	–		–	15	2	–
Ireland	–	–	–		–	–	–	–		–	10	–	–
Lithuania	–	–	–		–	–	–	–		–	5	–	–
Portugal	–	–	–		–	–	–	–		–	1	–	–
Slovenia	–	28	–		–	–	–	–		–	41	–	–
Switzerland	–	9	–		–	–	–	–		–	87	–	–
Luxembourg	–	–	–		–	–	–	–		–	–	4	–
Totals	230	487	1,034,114		13	5	1	364,727		1,116	1,565	64	8,058,831
Total infected	717				19					2,745			

*Table includes all reported HPAI H5N8 outbreaks through July 31, 2017. It excludes the new wave of secondary H5N8 outbreaks observed in Italy from the beginning of July 2017 through September 2017, which has different drivers and kinetics with maintenance in the poultry (primarily turkey) population rather than through wild bird introduction. FYROM, the former Yugoslav Republic of Macedonia; HPAI, highly pathogenic avian influenza.  †It is uncertain if for some outbreaks only the number of poultry in one farm building or if the poultry population in the area of the farm were reported. This estimate should be used as an approximation and indicator of impact. ‡One observation contained 600,000 birds, representing the overall population of backyard flocks affected in Romania. This number is an approximation. §These countries did not submit data to the Animal Disease Notification System in 2005–06; however, there is other evidence of H5N1 incursion in the period.

The number of wild bird detections was substantially different between epizootics: 1,559 incidents in 27 countries in 2016–17, 487 in 18 countries in 2005–06, and only 5 in 3 countries in 2014–15. Almost half of the wild bird incidents reported in all 3 epizootics were in Germany.

### Wild Birds Species and Mass Mortality Events

A total of 49 different wild bird species were reported infected with HPAI H5 virus of the Guangdong lineage in 2016–17, 28 in 2005–06, and 6 in 2014–15 (Table 2,3). Swans (*Cygnus* spp.), particularly mute swans (*Cygnus olor*), were the most frequent species infected in 2005–06 (41% of all wild birds) and 2016–17 (20% of all wild birds). Ducks were the second most common type of wild birds infected. In 2005–06 and 2016–17, tufted duck (*Aythya fuligula*) was the most frequent duck species detected positive (5% of all wild birds). In 2005–06, a total of 28 (6%) mass mortality events (>5 birds dead in 1 location) were reported, whereas 112 (7%) mass mortality events were reported in 2016–17; none were reported in 2014–15 (Technical Appendix Figure 2). The number of wild birds found dead by incident was significantly different between epizootics (p<0.001 by Mann-Whitney U test).

**Table 2:** Wild bird species of the orders Podicipediformes, Anseriformes, and Charadriiformes, reported by event in 3 highly pathogenic avian influenza epizootics in Europe

Species group	Species	No. (%) events		
		H5N1 2005–06 epizootic	H5N8 2014–15 epizootic	H5N8 2016–17 epizootic
Rails	Eurasian coot (*Fulica atra*)	5 (1)		8 (0.5)
	Crested coot (*Fulica cristata*)			1 (0.1)
	Purple swamphen (*Porphyrio porphyrio*)	4 (1)		
	Common moorhen (*Gallinula chloropus*)	1 (0.2)		2 (0.1)
	Total	10 (2)		11 (1)
Swans	Unspecified.	197 (38)	2 (22)	262 (16)
	Mute swan (*Cygnus olor*)	92 (18)		344 (20)
	Whooper swan (*Cygnus cygnus*)	2 (0.4)		80 (5)
	Total	291 (56)	2 (22)	683 (41)
Ducks	Unspecified	57 (11)		143 (9)
	Northern pintail (*Anas acuta*)	2 (0.4)		
	Eurasian wigeon (*Anas penelope*)		1 (11)	21 (1)
	Mallard (*Anas platyrhynchos*)	4 (1)	1 (11)	43 (3)
	Common pochard (*Aythya farina*)	4 (1)		8 (0.5)
	Red-crested pochard (*Netta rufina*)			2 (0.1)
	Common goldeneye (*Bucephala clangula*)			1 (0.1)
	Greater scaup (*Aythya marila*)	2 (0.4)		
	Common merganser (*Mergus merganser*)	5 (1)		
	Tufted duck (*Aythya fuligula*)	18 (3)		82 (5)
	Eurasian teal (*Anas crecca*)		1 (11)	3 (0.2)
	Smew (*Mergus albellus*)	1 (0.2)		
	Shelduck (*Tadorna tadorna*)			2 (0.1)
	Common eider (*Somateria mollissima*)			2 (0.1)
	Total	93 (18)	3 (33)	307 (18)
Geese	Unspecified	30 (6)		94 (6)
	Canada goose (*Branta canadensis*)			5 (0.3
	Barnacle goose (*Branta leucopsis*)	1 (0.2)		
	Greater white-fronted goose (*Anser albifrons*)			9 (1)
	Lesser white-fronted goose (*Anser erythropus*)	2 (0.4)		4 (0.2)
	Greylag goose (*Anser anser*)	1 (0.2)		21 (1)
	Red-breasted goose (*Branta ruficollis*)	1 (0.2)		
	Bean goose (*Anser fabalis*)			1 (0.1)
	Pink-footed goose (*Anser brachyrhynchus*)			1 (0.1)
	Total	35 (7)		134 (8)
Gulls	Unspecified	9 (2)		89 (5)
	Great black-backed gull (*Larus marinus*)			11 (1)
	Herring gull (*Larus argentatus*)	1 (0.2)		28 (2)
	Black-headed gull (*Larus ridibundus*)	1 (0.2)	1 (11)	23 (1)
	Lesser black-backed gull (*Larus fuscus*)			1 (0.1)
	Common gull (*Larus canus*)			2 (0.1)
	Total	11 (2)	1 (11)	154 (9)
Waders	Green sandpiper (*Tringa ochropus*)			1 (0.1)
	Eurasian curlew (*Numenius arquata*)			1 (0.1)
	Total			2 (0.1)

**Table 3:** Wild bird species of orders other than Podicipediformes, Anseriformes, and Charadriiformes reported by event in 3 highly pathogenic avian influenza epizootics in Europe

Species group	Species	No. (%) events		
		H5N1 2005–06 epizootic	H5N8 2014–15 epizootic	H5N8 2016–17 epizootic
Birds of prey	Unspecified	30 (6)		
	Buzzard	1 (0.2)		6 (0.4)
	Eagle			1 (0.1)
	Falcon	1 (0.2)		3 (0.2)
	Hawk	1 (0.2)		3 (0.2)
	Owl.	2 (0.4)		4 (0.2)
	Barn owl (*Tyto alba*)	1 (0.2)		
	Peregrine falcon (*Falco peregrinus*)	1 (0.2)		8 (0.5)
	White-tailed eagle (*Haliaeetus albicilla*)			24 (1)
	Common buzzard (*Buteo buteo*)	7 (1)		70 (4)
	Rough-legged buzzard (*Buteo lagopus*)	1 (0.2)		
	Eurasian eagle-owl (*Bubo bubo*)	2 (0.4)		1 (0.1)
	Eurasian sparrowhawk (*Accipiter nisus*)			1 (0.1)
	Common kestrel (*Falco tinnunculus*)			2 (0.1)
	Northern goshawk (*Accipiter gentilis*)			1 (0.1)
	Total	47 (9)		124 (7)
Crows	Unspecified	1 (0.2)		
	Eurasian magpie (*Pica pica*)	1 (0.2)		4 (0.3)
	Hooded crow (*Corvus cornix*)			3 (0.2)
	Rook (*Corvus frugilegus*)			2 (0.1)
	Carrion crow (*Corvus corone*)			1 (0.1)
	Common raven (*Corvus corax*)			1 (0.1)
	Total	2 (0.4)		11 (1)
Grebes	Great crested grebe (*Podiceps cristatus*)	7 (1)		12 (1)
	Little grebe (*Tachybaptus ruficollis*)	1 (0.2)		4 (0.2)
	Total	8 (2)		16 (1)
Thrushes	Blackbird (*Turdus merula*)			1 (0.1
	Song thrush (*Turdus philomelos*)			2 (0.1)
	Total			3 (0.2
Pigeons, doves	Wood pigeon (*Columba palumbus*)			2 (0.1)
	Collared dove (*Streptopelia decaocto*)	1 (0.2)		1 (0.1)
	Rock dove (*Coumbia livia*)		1 (11)	
	Total	1 (0.2)	1 (11)	3 (0.2)
Herons	Unspecified	2 (0.4)		16 (1)
	Grey heron (*Ardea cinerea*)	4 (1)		48 (3)
	Total	6 (1)		64 (4)
Storks	Unspecified	2 (0.4)		
	White stork (*Ciconia ciconia*)			3 (0.2)
	Total	2 (0.4)		3 (0.2)
Pelicans	Unspecified. (*Pelcanus* spp.)			2 (0.1)
Terns	Common tern (*Sterna hirundo*)			2 (0.1)
Cormorants	Great cormorant (*Phalacrocorax carbo*)	6 (1)		17 (1)
Other	Unspecified	9 (2)	2 (22)	140 (8)

### Type of Poultry Farm and Clinical Manifestations

The types of poultry infected in each epizootic are shown in Table 4. In 2016–17, a large proportion of infected farms (40%) kept ducks. In 2005–06, many affected backyard flocks in Romania (176/230, 77%) had <100 birds, whereas 70% (9/13) of poultry farms infected in 2014–15 had >10,000 birds and >60% in 2016–17 had >1,000 birds (difference in flock size distribution, p<0.001 by Kruskal-Wallis test). When we excluded Romania from the comparison of flock size, there was no statistical difference in flock size between 2005–06 and 2016–17 (Technical Appendix Figure 3). 

**Table 4:** Types of poultry on infected farms in 3 highly pathogenic avian influenza epizootics in Europe*

Type of poultry	H5N1 2005–06 epizootic			H5N8 2014–15 epizootic			H5N8 2016–17 epizootic	
	No. (%) farms	No. with only 1 species		No. (%) farms	No. with only 1 species		No. (%) farms	No. with only 1 species
Ducks				3 (23)	0		495 (44)	433
Geese							113 (10)	81
Ducks and geese	29 (13)	0						
Turkey	5 (2)	1		3 (23)	0		91 (8)	82
Broilers	23 (10)	17		4 (31)	0		93 (8)	48
Laying hens							47 (4)	29
Pigeons							9 (1)	1
Guinea fowl							10 (1)	1
Peacocks							2 (0)	0
Pheasants							8 (1)	5
Quail							2 (0)	1
Ostrich							1 (0)	0
Backyard†	176 (77)	NA						
Unknown				2			360(32)	NA
Total infected farms	230			13			1,116	

*NA, not available. †Backyard represents those households that keep few birds, normally layer hens, for their own consumption. The category was used only in the 2005–06 epizootic.

**Figure 1:**
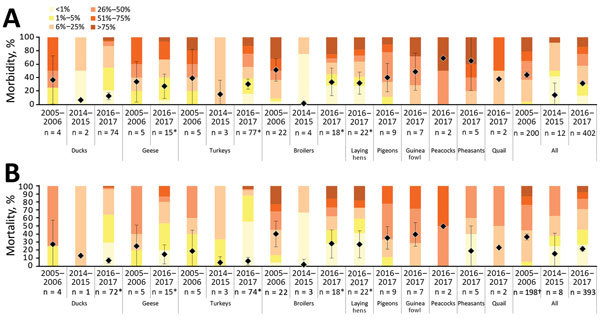
Morbidity (A) and mortality (B) rates as percentages of populations reported in infected poultry farms during 3 highly pathogenic avian influenza epizootics in Europe, 2005–06, 2014–15, and 2016–17. Years given are epidemiologic years (October through September of the next year). Diamonds with error bars indicate means and 95% CIs. Asterisks indicate farms with unique poultry species used for analysis; dagger indicates large majority of data from backyard farms reported in Romania.

Ducks, geese, turkeys, and broiler chickens on average had higher illness rates in 2005–06 than in the other epizootics (Figure 1). In 2016–17, average mortality rate was lowest in ducks (7%) and turkeys (6%); few farms (<5%) reported a >25% mortality rate. In contrast, 32% of affected broiler farms and 27% of affected layer farms reported mortality rates >25%. In 2005–06, more than half of broiler farms reported mortality rates >25%. When comparing overall estimates, we found the observed poultry illness and death rates to be substantially higher in 2005–06 than in 2016–17.

### Temporal Spread

**Figure 2:**
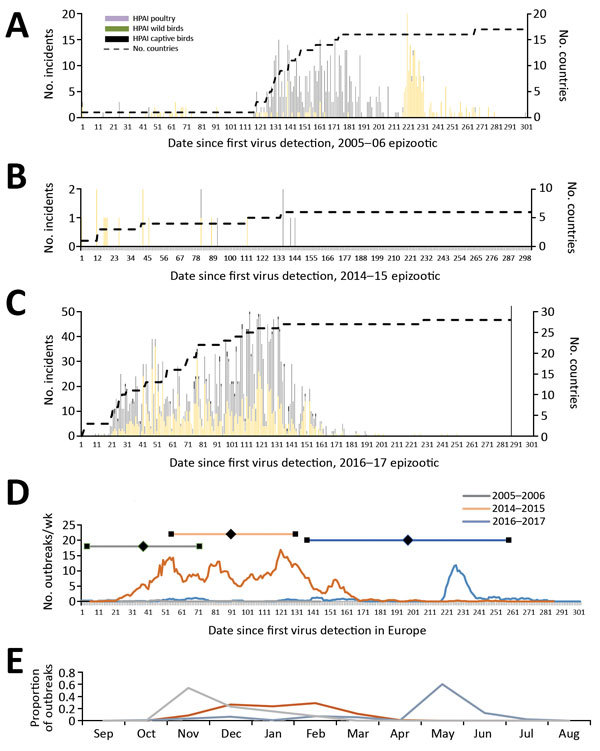
Epidemic curve of 3 HPAI H5 virus epizootics in Europe: A) 2005–06 H5N1; B) 2014–15 H5N8; C) 2016–17 H5N8. Years given are epidemiologic years (October through September of the next year). Dashed lines indicate number of countries reporting an HPAI infection since the beginning of the epizootic; vertical line in panel C indicates data collected through July 31, 2017. D) Weekly average number of poultry outbreaks for each epizootic. Horizontal lines indicate mean the day at which half of the poultry outbreaks have occurred (diamonds); error bars indicate 1 SD. E) Number of poultry outbreaks for each month for the 3 epizootics. HPAI, highly pathogenic avian influenza.

We determined the epidemiologic curves of the 3 epizootics (Figure 2, panels A–C). In 2016–17, H5 was first detected in Europe in a mute swan in Hungary; the first outbreak in poultry was detected 11 days later in a turkey farm, also in Hungary. We observed 3 major epidemic peaks on the incidence of poultry outbreaks (Figure 2, panel D): on day 54 (14.9 outbreaks/wk), following large farm-to-farm spread in Hungary; day 79 (12.1 outbreaks/wk) caused by farm-to-farm transmission in France and Bulgaria; and on day 121 (16.9 outbreaks/wk), caused by the large farm-to-farm spread in France and Poland.

In 2005–06 and 2016–17, a peak in wild bird incidents preceded the peak in poultry outbreaks (Figure 2, panel A, C). Statistical analysis of the distribution of the epidemic curves indicates that the 2016–17 outbreak had significantly higher incidence values (p<0.001 by 2-sample Kolmogorov-Smirnov test) than the other 2 epizootics; 2005–06 had significantly higher values (p<0.001 by 2-sample Kolmogorov-Smirnov test) than 2014–15. Temporal median of the poultry epizootic was substantially different between epizootics (mean/median distance for 2005–06, 189/223 days; for 2014–15, 33.5/26; for 2016–17, 92/90 days). Seasonal analysis of poultry outbreaks indicates significant differences (p<0.001 by Pearson χ^2^ test) between epizootics; >50% of poultry outbreaks occurred in May in 2005–06, in November in 2014–15, and in December–February in 2016–17 (Figure 2, panel E).

### Spatial Spread

**Figure 3:**
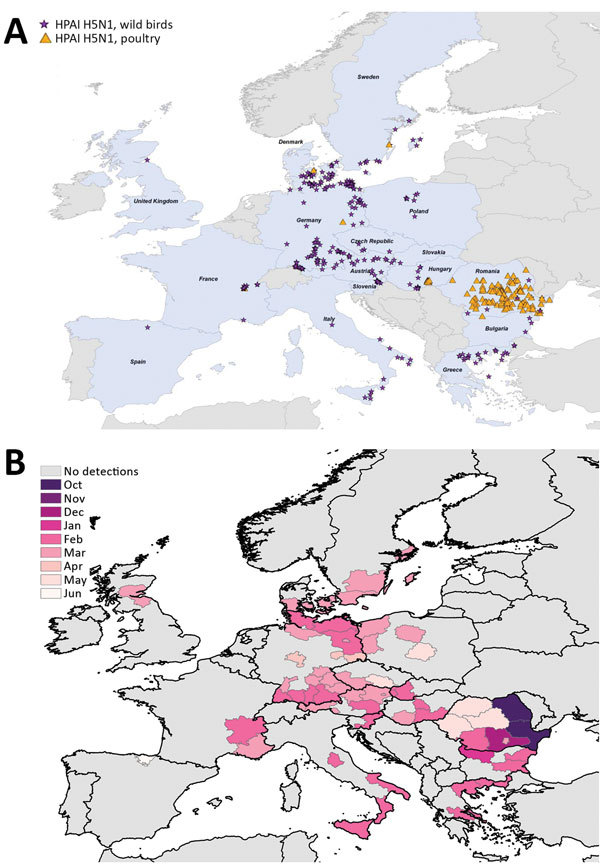
Geographic and temporal spread of the 2005–06 HPAI H5N1 epizootic. A) Location of each incident reported. Blue shading indicates countries where cases were reported. B) Month of first report of an HPAI H5N1 incident. Years given are epidemiologic years (October through September of the next year). HPAI, highly pathogenic avian influenza.

**Figure 4:**
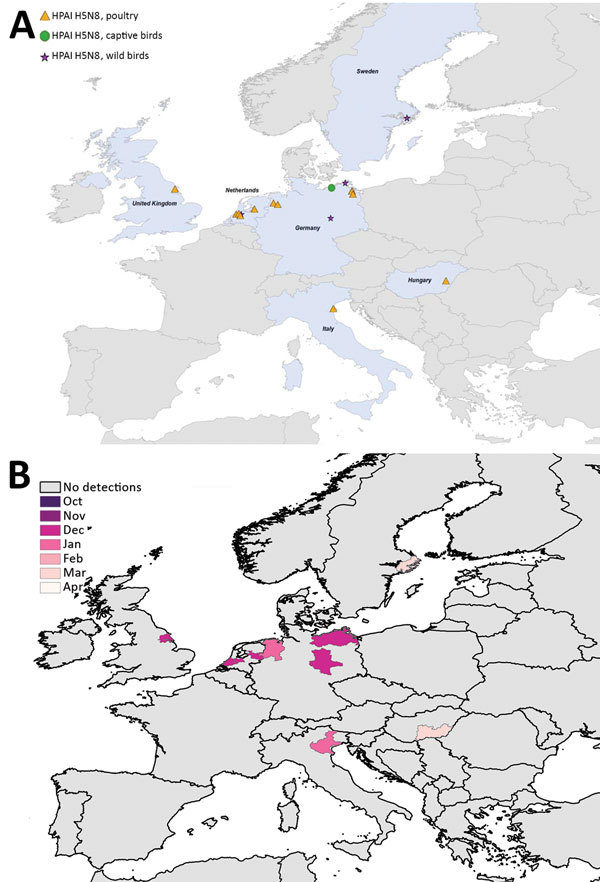
Geographic and temporal spread of the 2014–15 HPAI H5N8 epizootic. A) Location of each incident reported. Blue shading indicates countries where cases were reported. B) Month of first report of an HPAI H5N8 incident. Years given are epidemiologic years (October through September of the next year). HPAI, highly pathogenic avian influenza.

**Figure 5:**
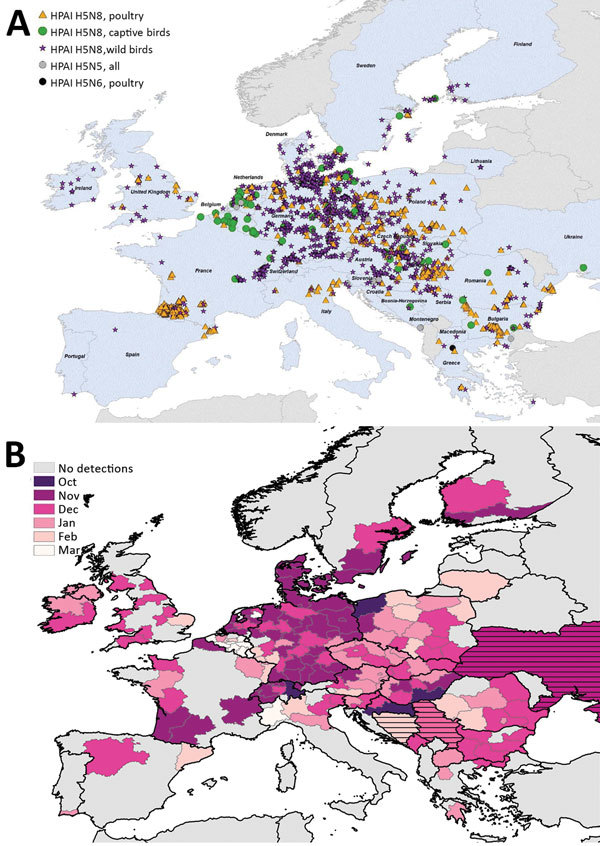
Geographic and temporal spread of the 2016–17 HPAI H5N8 epizootic. A) Location of each incident reported. Blue shading indicates countries where cases were reported. B) Month of first report of an HPAI H5N8 incident. Years given are epidemiologic years (October through September of the next year). HPAI, highly pathogenic avian influenza.

We mapped a temporal-spatial analysis of the 3 epizootics (Figures 3,4,5). The data shown in Figure 5, panel B, suggest that, in the first 2 months of the 2016–17 epizootic, 2 different viral incursions may have occurred: one spreading through Hungary, Croatia, Switzerland, and southern Germany, and another spreading in northern Europe (Poland, Denmark, northern Germany, Sweden, and the Netherlands). The 2005–06 epizootic indicated a similar progression pattern, initiating in Romania and spreading up to northern Europe and down to southeastern Europe (Figure 3).

Comparison by region of Europe according to wild bird migratory patterns indicates poultry outbreaks were mostly observed in the South-East and South-West regions in 2005–06 and 2016–17 but in the North in 2014–15 (Technical Appendix Figure 4). Most wild bird detections were reported in the North and Central regions. Poultry detections by region were significantly different for the 3 epizootics (p<0.001 by Pearson χ^2^ test), whereas wild bird detections by region were only significantly different (p<0.001 by Pearson χ^2^ test) between 2005–06 and 2016–17.

### Phylogenetic Analysis

**Figure 6:**
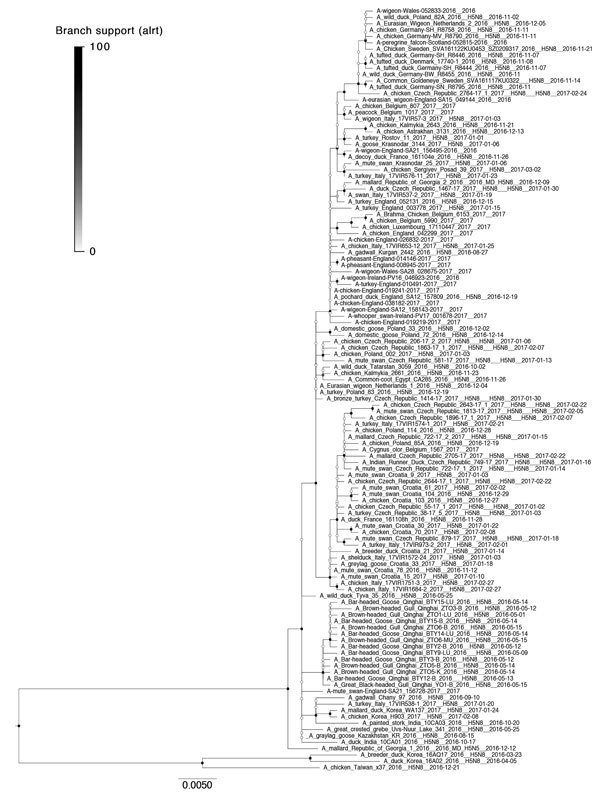
Maximum-likelihood tree from viral sequences of the 2016–17 highly pathogenic avian influenza H5 epizootic in Europe. Circles represent node support values, filled according to approximate likelihood ratio test values 0–100. Light gray boxes indicate distinct clades with support >50 with isolates from Europe; dark gray boxes indicate clades with <50 or unresolved. Scale bar indicates nucleotide substitutions per site. An expanded figure showing trees for all 3 epizootic years is available online (https://wwwnc.cdc.gov/EID/article/24/12/17-1860-F6.htm).

Genetic analysis of the HA gene for the 2014–15 and 2016–17 epizootics shows the involvement of H5 clade 2.3.4.4 in all cases where data were available (Figure 6). Patterns found in maximum-likelihood trees are largely in agreement with the Bayesian analysis; however, a greater proportion of the clades remain unresolved in the maximum-likelihood trees (Figure 6; Technical Appendix Figure 8). The 2016–17 viruses form a distinct clade and can be clearly differentiated from the clade 2.3.4.4 viruses present in Europe in 2014–15. In agreement with the geospatial results, analysis of the HA gene of the viruses from the 2016–17 epizootic shows that most originate from a common progenitor (time to most recent common ancestor estimated May 2014–August 2015) (Technical Appendix Figure 8). However, these viruses differ in their evolutionary pathway thereafter, evolving in 2 co-circulating subclades without clear geographic restriction (time to most recent common ancestor March 2015–August 2016 [0.9 posterior probability] and November 2014–October 2015 [0.82 posterior probability]). This finding potentially indicates 2 major incursion pathways via wild birds. 

We also found smaller clusters and singleton sequences including sequences from European viruses; viruses from 2014–15 form 1 subclade, estimated to have emerged in January–February 2014 (Figure 6; Technical Appendix Figure 8). The 2005–06 data show viruses in several subclades, but the branching pattern in this dataset is generally less distinct and many sequences remain unresolved.

BEAST analyses (http://tree.bio.ed.ac.uk/software/beast/) also revealed that the 2014–15 epizootic viruses show the highest mean substitution rate (measured per site per year), followed by 2016–17 and then by the 2005–06 epizootic, which is significantly lower (one-way analysis of variance p <0.001) (Technical Appendix Figure 6). These data are in agreement with the results of the root-to-tip regression analysis (Technical Appendix Figure 7), which show a much steeper slope for the 2014–15 epizootic compared with the others. However, the spread of the data is high for the 2016–17 epizootic, where the SD of rates is an order of magnitude higher than that for the 2014–15 epizootic and 2 orders greater than for the 2005–06 outbreak. The nucleotide diversity for each epizootic (Technical Appendix Figure 9) shows that per-site diversity (average pairwise nucleotide differences in a population) is lowest in the 2005–06 epizootic (0.0038), consistent with the lower substitution rate inferred from BEAST. The 2014–15 epizootic has the highest diversity (0.0086); the rate for 2016–17, calculated from viruses collected through June 2017, is 0.0063.

## Discussion

The 2016–17 epizootic of HPAI H5 clade 2.3.4.4 viruses in Europe has 5 times more outbreaks in poultry than observed in the H5 clade 2.2 epizootic in 2005–06 and 80 times more than in the H5 clade 2.3.4.4 epizootic in 2014–15. This study highlights the unprecedented magnitude of the 2016–17 HPAI H5 epizootic in Europe, in terms of size (both number of poultry outbreaks and wild bird incidents), geographic spread, speed of incidents/outbreaks, and diversity of wild bird species reported infected. As a result, the economic impact is many times higher for 2016–17, which resulted in an >8-fold increase in poultry that died or were culled.

A greater passive surveillance effort to detect influenza virus in wild birds was reported in the EU in 2006 than in 2016 (*20*,*21*). Despite reduced passive surveillance efforts in recent years, more virus detections were made in wild birds in calendar year 2016 compared with 2006, indicating a likely increase in viral burden within bird populations in Europe, leading to an increased risk for incursion into poultry. Although we found a lower rate of substitution and diversity in 2016–17 compared with 2014–15, the viruses in the 2016–17 epizootic might be more efficient in capacity to adapt and infect avian hosts. Different rates and diversity between 2005–06 and the 2 more recent epizootics may be caused by overall differences in the H5 lineages (clade 2.2 versus 2.3.4.4), which could influence viral spread. The greater genetic distances we observed in viruses detected in the 2014–15 epidemic could also be due to lower sensitivity of surveillance for this virus compared with the other 2 epidemics due to an apparently lower mortality rate in wild birds.

Extensive secondary spread is the most probable explanation for the large number of outbreaks reported in the farmed duck sector in 2016–17, possibly because of rapid attenuation of viral symptoms. Hence, on several farms with clinically healthy birds, we detected HPAI infections through active epidemiologic tracings and not on the basis of clinical signs, as reported in data from some member states. The results may also indicate that infection and transmission between domestic ducks is relatively easy for these viruses. The type of husbandry practices and frequent movement of birds, coupled with poor biosecurity and lack of robust hygiene practices, may also make the spread of the viruses between farms easier (*22*).

Swans and ducks were the predominant hosts infected in 2005–06 and 2016–17. Of interest, although mallards (*Anas platyrhynchos*) are the most frequently tested in EU passive surveillance (*4*), tufted ducks (*Aythya fuligula*) were the most commonly identified species of duck with HPAI in 2005–06 and 2016–17. In addition, the 2016–17 epizootic demonstrated a much expanded wild bird host range compared with previous outbreaks. In light of these results, we recommend a review of the target species for avian influenza surveillance (*5*) to improve sensitivity of surveillance. Clarifying the precise origins of the current epizootic viruses from reported wild bird mortality data is problematic, because these data do not allow distinction between migratory carrier species and resident sentinel species. Many of the reported species are not migratory (e.g., mute swan or little grebes) and so might play a role as regional amplifiers of viruses but not in long-distance spread (*23*).

Epidemic curves for the 3 epizootics were significantly different. The incidence values in order of magnitude were 2016–17 > 2005–06 > 2014–15. In the period of the review, the mean temporal distances to the midpoint in the poultry epizootic were different; 2014–15 was relatively short, consistent with the incursion into the poultry sector and potentially lower virus infectivity present in the wild bird reservoir, whereas in 2005–06 and in 2016–17, epidemic curves show a clear peak of detection of wild bird incidence preceding the peak of poultry incidences, which demonstrates the importance of wild bird surveillance.

For the 2016–17 epizootic, the epidemic curve shows a long extended tail with small sporadic peaks relating to localized but limited detection and spread in both poultry and wild birds (Figure 2, panel C). These data might suggest greater infection pressure from migratory birds in 2016–17, leading to higher risks for incursion, greater environmental contamination, and exposure of local indigenous wild bird populations and poultry. The observed spatiotemporal relationships between poultry incursions and wild bird detections represent a complex dynamic. Exploration of the epidemic curves by country in 2016–17 shows important differences that relate to the type of poultry production infected (Technical Appendix Figure 7). For example, we detected infections in Hungary relatively early in the epizootic; their rapid peak and decline may reflect extensive infection within the major duck-producing regions and less susceptible populations through infection and depopulation. In contrast, infection in Germany and Poland was more consistent and may reflect a more continuous exposure and incursion risk into a variety of poultry sectors.

The viruses showed close genetic similarity to viruses contemporaneously circulating in Central and Southeast Asia. The lower genetic diversity observed in 2016–17 was accompanied by reassortment of all gene segments, as shown in previous studies (*8*,*24**,**25*). The high reassortment observed in the 2016–17 epizootic also resulted in novel NA reassortants such as the H5N6 and H5N5 viruses. The H5N6 viruses circulating in Europe were a reassortant of HPAI H5N8 and classical European LPAI present in wild birds (data not shown). We can clearly differentiate the genetic characteristics of this strain from viruses known to be circulating in poultry and wild birds in the Far East with occasional spillover to humans.

Epidemiologic results suggest 2 broad corridors of virus incursion in 2005–06 and 2016–17, through northern and central Europe with subsequent spread, later corroborated through phylogenetic analyses of the HA gene of the viruses from the 2016–17 epizootic. This dual incursion probably relates broadly to known postbreeding movements of northern duck species, which breed widely across northern Eurasia (*11*,*13*,*26*). These movements occur on a broad front, but ringing recoveries and other analyses demonstrate movements from breeding areas from Siberia both southwest toward the Black and Aegean Seas and ultimately the coastal wetlands of the eastern Mediterranean, and further north and west through the Baltic Sea to coastal and other wetlands of the southern North Sea and northwestern countries (*11*–*14*). These represent migratory tendencies only; several studies have shown the high-level complexity of these movements and their variation due to both short-term weather patterns and longer-term climate change (*27**,**28*). The fact that these corridors were apparent in 2 temporally distant epizootics suggests the need for further research to focus surveillance in these areas.

This study presents many limitations (online Technical Appendix). Differences in the implementation of passive wild bird surveillance between countries, which are implied in the EU avian influenza annual report for 2016 (*20*), suggest that sensitivity of wild bird surveillance varies across countries (*29*), which could affect the distribution of cases we observed. The true probability of detecting HPAI is dependent on many factors that may influence both the frequency of wild bird deaths and the likelihood of identification and sampling of wild bird carcasses in different regions and countries. Public awareness, the current avian influenza status of the country area, media coverage, prevailing climatic conditions, available food sources, and removal by predators may affect wild bird mortality, detection rates, or both (*30*). Furthermore, the efficacy of passive surveillance is difficult to measure because capturing the expended effort depends on observation and testing of deceased birds. On the other hand, surveillance has high sensitivity in farmed poultry, mainly because of higher virulence and much closer observation of these populations.

Despite apparent heavy infection pressure in wild birds in 2016–17, the virus was not detected early in the epizootic in areas in eastern Europe, such as the Danube Delta, with high density of early migratory waterfowl. There were significant incursions in poultry in northern Europe, particularly Germany and Poland, and these areas also reported the greatest number of infected wild birds. This finding may reflect the implementation of enhanced surveillance in wild bird populations rather than true increased risk. Southwestern Europe had relatively few wild bird detections compared to the number of poultry outbreaks, perhaps because of the establishment of the virus in the duck production sector in southwestern France, not as a result of increased introductions from wild birds (*31*).

The extent of the 2016–17 H5 epizootic indicates an urgent need to reappraise the effectiveness of surveillance strategies in both wild and domestic birds and to monitor key populations for emergence of viral variants. The differences we observed in the 3 epizootics illustrate the difficulty of predicting HPAI epizootics. However, the temporal peak of wild bird detections preceding the peak of poultry outbreaks at the EU level highlighted the utility of surveillance in wild birds, as observed in other studies (*29*). The spatial corridors of HPAI we identified may provide the basis for an increase in targeted surveillance to improve system sensitivity. Although the H5N8, H5N5, and H5N6 European-reassortant viruses have not been shown to infect humans and remain avian influenza–like strains with no evidence of key mammalian adaptation markers (*27*), their genetic volatility represents a potential threat that requires continuous monitoring and surveillance of virus incidence and genetics to continue to protect public safety.

Technical Appendix Additional information about 3 epizootics of highly pathogenic avian influenza H5 Guangdong lineage in Europe.
