# Supplementary material for: Comparison of 2016–17 and Previous Epizootics of Highly Pathogenic Avian Influenza H5 Guangdong Lineage in Europe
Source: Emerg Infect Dis. 2018 Dec;24(12):2270–83. doi: 10.3201/eid2412.171860 (PMC6256410; doi:10.3201/eid2412.171860)
Supplement: Technical Appendix — Additional information about 3 epizootics of highly pathogenic avian influenza H5 Guangdong lineage in Europe. [file 17-1860-Techapp-s1.pdf]

Article DOI: https://doi.org/10.3201/eid2412.171860

Comparison of 2016–17 and Previous Epizootics of Highly Pathogenic Avian Influenza H5 Guangdong Lineage in Europe

Technical Appendix

Section A1. Materials and Methods

A1.1. Epidemiologic Analysis

Data were collected from the 3 major HPAI H5 epizootics in Europe: the HPAI H5N1 epizootic in 2005 and 2006 (“2005–06”); the HPAI H5N8 epizootic in 2014 and 2015 (“2014–15”); and the HPAI H5 epizootic in 2016 and 2017 (“2016–17”). For the 2016–17 epizootic, records were collected between October 19, 2016 and July 31, 2017. Epidemiologic data were obtained from the Animal Disease Notification System (ADNS), managed by the European Commission, DG SANTE (Directorate for Health and Food Safety), which is the official platform for EU MS to report outbreaks of notifiable animal diseases; and from country notifications sent to the EU Reference Laboratory for Avian Influenza (EURL – Animal and Plant Health Agency, Weybridge, UK).

The following data fields were collated: suspicion date, confirmation date, country, geographic coordinates, bird type (poultry, wild bird, or captive bird), bird species, number of birds at risk on the farm, total cases, total deaths, and total animals destroyed. Data available on the ADNS system may be subject to differences in interpretation from system users. Biases, data gaps, and uncertainty in the data uploaded to the ADNS system are listed in the section **Biases associated with ADNS data** in section A1.3 of this appendix.

All confirmed cases were identified from samples tested using recommended diagnostic tests (Commission Decision 2006/437/EC) as described in the OIE manual of diagnostic tests (EC 2006) (*1*).

All statistical analyses were performed in STATA 14 (StataCorp, College Station, TX, USA).

Epidemiologic analyses were done to:

• ***Describe the size of the epizootics:*** frequency of poultry outbreaks and wild bird incidents by country and poultry type or wild bird species were described.

• ***Investigate differences in geographic spread:*** spatial analyses were performed using ArcMap 10.2.2 (ESRI, USA) to visualize locations of poultry outbreaks and wild bird incidents, and to assess spatial progression of the epizootics by month. In addition, geographic spread was examined in relation to major wild bird migratory patterns. Countries were grouped into 4 regions based on the broad migration patterns of the major migratory water bird species affected by HPAI (Technical Appendix Figure 1). Justification for these groupings can be found under the heading **Derivation of geographic regions** in section A1.4 of this appendix. The regions were defined as follows:

- North of Europe: Belgium, Denmark, Estonia, Finland, Latvia, Lithuania, Luxembourg, Ireland, North France (above 45 latitude), North Germany (above 50 latitude), Norway, Poland, Sweden, The Netherlands, and United Kingdom.
- South-West Europe: Croatia, Malta, Italy, Portugal, Slovenia, South of France (below 45 latitude), and Spain.
- South-East Europe: Bulgaria, Cyprus, Greece, Hungary, Serbia and Romania.
- Central Europe: Austria, Czech Republic, Slovakia, South Germany (below 50 latitude), and Switzerland.

• ***Investigate differences in temporal spread:*** We compared the different epidemic curves. We tested for differences in the shape of epidemic curves using a 2-sample Kolmogorov–Smirnov test to measure the temporal distance to the median point of the poultry epizootic (day at which half of poultry outbreaks have occurred). In addition, seasonality was compared (period of the year where cases have occurred).

• ***Investigate differences in clinical presentation:*** Differences in the number of mass die-off events observed in wild bird species were tested as a measure of disease impact in wild birds.

Differences in clinical presentation in poultry were tested by comparing poultry morbidity and mortality data for each species in each epizootic. Estimation on morbidity using ADNS data was used from farms rearing only 1 species when possible (e.g., only ducks). Morbidity was calculated based on 3 variables collected on ADNS:

- At risk = number of animals at risk of infection on the farm at time of investigation
- Cases = number of cases observed on the farm at time of investigation
- Deaths = number of bird dead on the farm at time of investigation

Due to different system users inputting data to the ADNS databank, it is suspected that while most observations in the "At risk" data field correspond to the total number of birds on the farm before the outbreak (deceased, moribund and healthy birds), in some instances this field may have been interpreted to represent the number of healthy animals remaining on the farm, or the total number of animals within an epidemiologic unit on the farm. For the "Cases" data field, it is suspected that some entries include both deceased and moribund birds within the estimation, while some entries only report moribund birds, alive with clinical signs. The following assumptions were made for the calculation of morbidity:

- Number of "At risk" was believed to be the total number of animals on the farm.
- The "Cases" field includes both the number of dead and moribund birds, except in those entries where the number of deaths is larger than number of cases (74 entries, which is 9.3% of poultry morbidity estimates). When the number of deaths is bigger than number of cases, the total number of cases was believed the sum of "cases" and "deaths."
- When the "Cases" field was blank, and the number of "Deaths" was known, the number of deaths was used to represent the number of cases observed on the farm (n = 265, which is 33.4% of morbidity estimates).
- All farms with null "Cases" and "Deaths" were discarded, as these were considered to be farms without clinical disease, reported as dangerous contacts due to links with infected farms.

A1.2. Phylogenetic Analysis

Virus haemagglutinin (HA) gene sequence data were obtained from countries' submissions to EURL; sequences generated as part of this study; and from the Global Initiative on Sharing All Influenza Data (GISAID) platform downloaded on June 2, 2017. To determine the genetic relationships among strains circulating in each epizootic, we analyzed viral sequence data from each outbreak separately. Haemagglutinin (HA) gene sequences of viruses from each epizootic were first subject to a quality control step where all duplicate sequences, sequences with <900 nt, and those with >10% undefined nucleotides (Ns) were discarded. Sequences in each dataset were aligned using MAFFT v7.305b, and trimmed to remove signal sequence at the N terminus and STOP codon at the C-terminus. Trimmed sequences were realigned using prank v.170427 with model parameters codon (empirical codon model) and F (force insertions to always be skipped).

IQ-TREE version 1.5.5 was used to infer maximum-likelihood trees from each dataset. The codon model was determined by the Model Finder algorithm within IQ-TREE, and both alrt (approximate likelihood ratio test, 1000 replicates) and bootstrap (100 replicates) were calculated to determine support for branching. The "best tree" was then annotated in FigTree v1.4.3.

Then, each dataset was down-sampled using CD-HIT-EST (Cluster Database at High Identity with Tolerance) to remove sequences with >99.9% sequence identity. The down-sampled datasets contained 244 (2005–6), 134 (2014–15) and 91 (2016–17) sequences.

Root-to-tip regression analyses were performed using Tempest v1.5 on the down-sampled datasets before Bayesian phylogenetic trees were inferred using BEAST v1.8.4 to determine the mean substitution rate and TMRCA (time to most recent common ancestor). The HKY site model was used with estimated base frequencies and gamma site heterogeneity with 4 gamma categories and 3 codon partitions. An uncorrelated relaxed lognormal clock was used with constant population prior and random starting tree. All priors were set to default except allMus which was set to a uniform distribution ranging from 0 to 1E100. MCMC was set to 100 million generations. Log files were analyzed in Tracer v1.6.0, to determine convergence, and check ESS values were beyond threshold (>200). Tree annotator v1.8.4 was used to generate a maximum credibility tree (MCC) using 10% burnin and median node heights. The MCC tree was

then annotated to include time scale and mean substitution rate in FigTree v 1.4.3. The nucleotide diversity for each outbreak was measured using the PopGenome package in R.

A1.3. Biases Associated with ADNS Data

The following data gaps and uncertainties were detected in the poultry data:

Biases related to notification date: Date of suspicion was missing for 24 outbreaks in 2005–06 and 319 outbreaks in the 2016–17 epizootics. Date of confirmation was used as the date of suspicion for the epidemic curves. It is possible that some countries have used the same confirmation date to report all incidents that have occurred within a week. This is possible due to the high work load pressure during the period with large number of outbreaks.

Biases related to morbidity and mortality estimates: Assumptions and uncertainties of the data have been described in section A1.1 of this online appendix. Number of cases and deaths were missing for many of the poultry outbreaks reported, which reduced the power of the analysis to detect differences. Information on cases were reported for 530 outbreaks (38% of all poultry outbreaks; 26% of outbreaks in 2005–06; 85% of outbreaks in 2014–15; and 40% of outbreaks in 2016–17). Information on number of deaths were reported for 776 outbreaks (56% of all poultry outbreaks; 86% of outbreaks in 2005–06; 62% of outbreaks in 2014–15; and 50% of outbreaks in 2016–17). When possible morbidity was estimated for farms having unique species, however this was not possible in all cases (e.g., Ducks in 2005–06). Morbidity and mortality estimates may be biased in some cases due to the existence of other poultry types on the premises (more or less susceptible to HPAI).

Biases related to flock size: Most observations in the “At risk” category should indicate number of birds reported on the farm, however it may be possible that some entries indicate only number of birds in one of the buildings of the farm.

Bias related to differences between countries: Variation in the poultry demographics between countries, regions within countries and over time may influence the detection of AI. Some of the variables implicated in this include: industry structure (high levels or organization and/or industry veterinarians may effect detection rates), industry engagement (aware of AI and proactive in reporting disease symptoms– or not), density of poultry systems (poultry farms clustered in close proximity), poultry types (anseriforme poultry types less likely to present

disease symptoms for detection), type of poultry production systems (indoor/outdoor/back yard flocks), variation in sampling strategies between countries and over time may lead to misrepresentation of the true distribution of AI poultry outbreaks.

Bias could also be introduced in the following ways: sensitivity of passive surveillance (procedures for detection by state vet services), intensity of scanning surveillance (serology), frequency of serology positive PCR follow-ups (e.g., sampling at slaughter influence proportion of PCR follow-ups?), and changes to intensity of surveillance following recent outbreaks in close proximity. Differences on implementation of passive surveillance in wild birds by countries are shown in the EU Avian Influenza surveillance report (*2*).

The following data gaps and uncertainties were detected in the wild bird ADNS data:

Biases related to "Notification date": "Date of suspicion" was missing for 1,191 wild birds incidents (76.4%) in the 2016–17 epizootics and date of confirmation was used as the date of suspicion for the epidemic curves. In addition, seven wild bird incidents had the same suspicion and confirmation date. It is possible that some countries have used the same confirmation date to report all incidents that have occurred within a week. This is possible due to work load pressure during the period with high number of outbreaks.

Biases related to number of wild birds death reported by incident: It is possible that for many incidents countries reported only those animals that they have tested and confirmed positive and did not report all the birds found dead in each incident.

Heterogeneity in wild bird species and density across space and time may lead to differences in detection rates in some of the following ways: density of wild birds combined with level of human traffic influence the likelihood of an individual coming across a sick/dead bird and reporting it, species of wild birds are heterogeneously distributed across Europe, some species are less likely to exhibit disease symptoms/suffer mortality, temporal variation in wild birds densities and species types due to seasonal migration.

Wild bird surveillance is carried out actively (sampling live healthy birds, mainly detecting LPAI H5 and H7) and passively (sampling sick or found-dead birds, mainly detecting HPAI). Each of these strategies may introduce bias in the following ways:

- Active surveillance of live healthy birds: There are differences in time and resources countries dedicate to the capture of live birds to sample. This influences the likelihood of finding infected birds, if present. This may be one reason for the high detection rate of infected wild birds in Germany. Active surveillance only samples birds in the target species list.
- Passive surveillance of sick/injured and birds found dead: Variety in state effort put into searching for dead wild birds to sample. Public awareness in reporting dead wild birds may be different between countries, Likelihood of detection of wild birds found dead may vary with species (e.g., big white birds easier to spot than little brown birds. Little birds scavenged more readily), Increase in local surveillance spatially and temporally during an outbreak

A1.4. Derivation of Geographic Regions

Each migratory waterbird species has a migration pathway that comprises a unique spatial-temporal combination of habitats used and geographic areas occupied. While typically there is high year-to-year consistency in migration patterns, many species show flexibility in occurrence related to the short-term influences of weather, as well as longer term consequences of changing climate.

Migration pathways for species of similar ecology have been aggregated into flyways – defined as: "A flyway is the entire range of a migratory bird species (or groups of related species or distinct populations of a single species) through which it moves on an annual basis from the breeding grounds to non-breeding areas, including intermediate resting and feeding places as well as the area within which the birds migrate" (*3*).

There is good knowledge of the flyways and areas of occupancy of most European waterbirds, summarized both in published atlases, for ducks, geese and swans (*4*) and waders (*5*), as well as online for other species (e.g., Wings over Wetlands).

To summarize geographically the broad occurrence of H5 virus outbreaks for this study in the context of migratory waterbird distribution, we assigned countries to one of 4 regions based on the combination of broad distribution and migration patterns of relevant duck, goose

and swan species (although informed by the distribution of other migratory waterbirds as relevant).

Movements of many ducks are similar, showing post-breeding movements from tundra and taiga habitats in western and central Russia, westwards to non-breeding areas in western and southern Europe (*4*,*6*,*7*). Recoveries of ringed birds and more recent new tracking technologies indicate population structuring: broadly, with two separate biogeographical populations occurring i) in northwest Europe (UK and Ireland, northern France, and countries surrounding the North and Baltic Seas); and ii) in the countries of the Mediterranean basin from Iberia to Israel, together with the Black Sea. This is the case for Eurasian Wigeon (*Anas penelope*), Gadwall (*Anas strepera*), Common Teal (*Anas crecca*), Common Pochard (*Aythya ferina*), and Tufted Duck (*Aythya fuligula*). For some species such as Mallard (*Anas platyrhynchos*), the southern wintering Mediterranean birds are split into western and eastern Mediterranean populations, while the population range of southern wintering Pintail (*Anas acuta*) and Northern Shoveler (*Anas clypeata*) also extends to west and sub-Saharan Africa. Other species, such as Greater Scaup (*Aythya marila*), other seaducks, and arctic-breeding geese occur around the North Sea basin without more southerly Mediterranean wintering areas.

The 4 regions defined in this paper (North, South-West, South-East, and Central Europe) aim to broadly categorize distinct geographic areas used by different biogeographic populations of waterbirds. For ease of data-handling, whole countries are assigned to different regions other than for France and Germany. Waterbirds using the coasts and inland wetlands of southern France were assigned to South-West Europe, while southern Germany was included in Central Europe.

Section A.2 – Comparative Statistical Analysis and Results

A2.1. Number of wild bird deaths reported by incident in the 3 epizootics

Results show that number of wild birds death reported by incident were different between the 2005–06 and 2016–17 epizootic ($p<0.001$) (Technical Appendix Figure 2).

A2.2. Distribution of poultry flock sizes of infected poultry premises between epizootics

Technical Appendix Figure 3 shows the distribution of poultry flock size in farms detected with H5 HPAI in the 3 epizootics.

A2.3. Frequency of poultry outbreaks and wild bird incidents by European region between the 3 epizootics

During the H5N1 HPAI outbreak of 2005–06 the majority of poultry detections were made in the South-East European region (98%), while detections were seen across all regions of Europe in the H5N8 HPAI outbreak 2016–17, with the majority of detections reported from South-East Europe and South-West Europe (Technical Appendix Figure 4). Bosnia and Herzegovina (n = 1), Serbia (n = 4), FYRO Macedonia (n = 1) and Ukraine (n = 2) were not included in the regions. The Pearson Chi-square test to compare the epizootics indicates that distribution of poultry outbreaks in the four regions does differ between epizootics ($p < 0.001$). Pearson Chi-square test indicate that distribution of wild birds incidents report in the 4 regions is also different between 2016–17 and 2005–06 epizootics ($p < 0.001$), but not significantly different with 2016–17 and 2014–15 ($p = 0.282$) and only weak evidence of difference between 2005–06 and 2014–15 ($p = 0.05$).

A2.4. Comparison of the epidemic curves between the 3 epizootics

Three types of analysis were done to assess differences in the distribution and values of the epidemic curve; differences in the distribution to the day at which half of poultry outbreaks have occurred; and difference in seasonality.

Differences in the distribution of the 3 poultry epidemic curves

A 2-sample Kolmogorov–Smirnov test to assess equality of distribution functions was performed. Results indicate that the 2016–17 epizootic contains significantly higher values than the 2005–06 ($p<0.001$) and 2014–15 ($p<0.001$) epizootics, and that the 2005–06 epizootic contains significantly higher values than the 2014–15 epizootic (p-value<0.001).

Differences in distance to the day at which half of poultry outbreaks have occurred

The distance (“mean distance”) was calculated using day 1 as the day when the virus was first detected in Europe, either in wild birds or poultry. Results indicate that half of poultry outbreaks were reported on day 198 in 2005–06 epizootic, on day 38 in the 2014–15 epizootic and on day 91 in the 2016–17 epizootic. T-test and Wilcoxon rank sum test were performed to assess for evidence of differences between mean distances of 2 epizootics. The results of the analysis show that distance were significantly different between the 3 epizootics.

Difference in seasonality of poultry outbreaks between epizootics

The frequency of poultry outbreaks per month were examined. Pearson Chi-square tests were performed to assess for statistical difference in the distribution of poultry outbreaks per month, using pairwise comparison between epizootics. Results indicate that the distribution are significantly different between the three outbreaks (p-value<0.001).

Epidemic curves in the most important countries in the 2016–17 HPAI H5N8 epizootic

Epidemic curve of the 2016–17 HPAI H5N8 epizootic in Europe for the 6 countries most affected by the epizootic are shown in Technical Appendix Figure 9.

Section A4. Additional Results Phylogenetic Analysis

Technical Appendix Figures 6–8 show additional results of the phylogenetic analysis conducted.

References

1. EC, Commission Decision 2006/437/EC of 4 August 2006 approving a Diagnostic Manual for avian influenza as provided for in Council Directive 2005/94/EC. Official Journal of the European Union 2006. **L 237**: p. 1.
2. European Union Reference Laboratory For Avian Influenza. Annual report on surveillance for avian influenza in poultry and wild birds in Member States of the European Union in 2016. European Union Reference Laboratory for Avian Influenza. European Commission. 2017 [cited 2018 Apr 27]. https://ec.europa.eu/food/sites/food/files/animals/docs/ad_control-measures_ai_surv-rslt_pltry-wld-brds_2016.pdf.
3. Boere G, Galbraith C, Stroud D; Scottish Natural Heritage. The flyway concept: what it is and what it isn’t. In: Waterbirds around the world: a global overview of the conservation, management, and research of the world's waterbird flyways, Edinburgh: The Stationery Office; 2006
4. Scott DA, Rose PM. Atlas of Anatidae populations in Africa and western Eurasia. Publication No. 41. Wageningen, The Netherlands: Wetlands International; 1996.
5. Delany S, Scott D, Dodman T, Stroud DA. An atlas of wader populations in Africa and western Eurasia. Wageningen, The Netherlands: Wetlands International; 2009.

6. Wernham CV, Toms M, Marchant JH, Clark J, Siriwardena G, Baillie S, editors. The migration atlas: movements of the birds of Britain and Ireland. London: T. & A.D. Poyser Ltd; 2002.

7. Viksne J, Švažas S, Czajkowski A, Janaus M, Mischenko A, Kozulin A, et al. Atlas of duck populations in eastern Europe. Vilnius, Lithuania: Oiseaux Migrateurs du Palearctique Occidental; 2010.

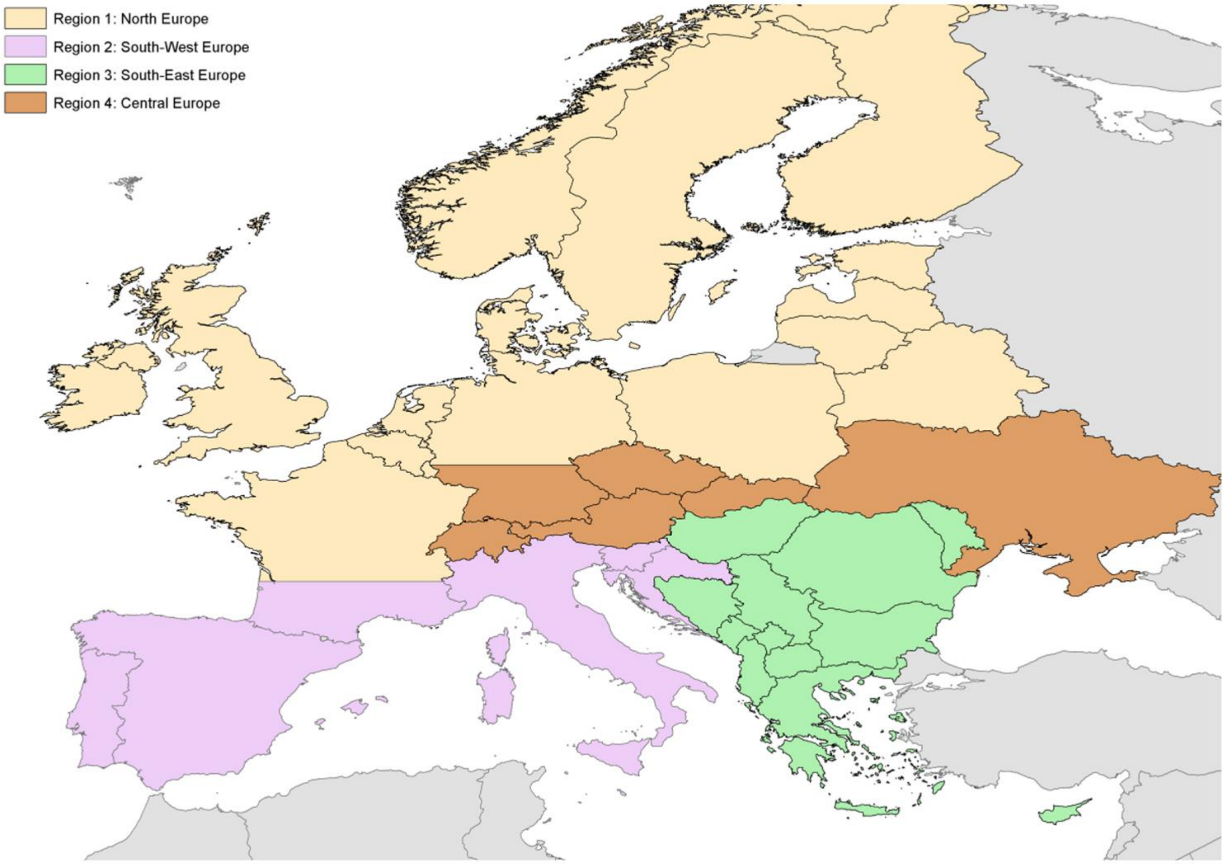

Technical Appendix Figure 1. Map of European regions based on wild bird migratory patterns, used for analysis of highly pathogenic avian influenza detections.

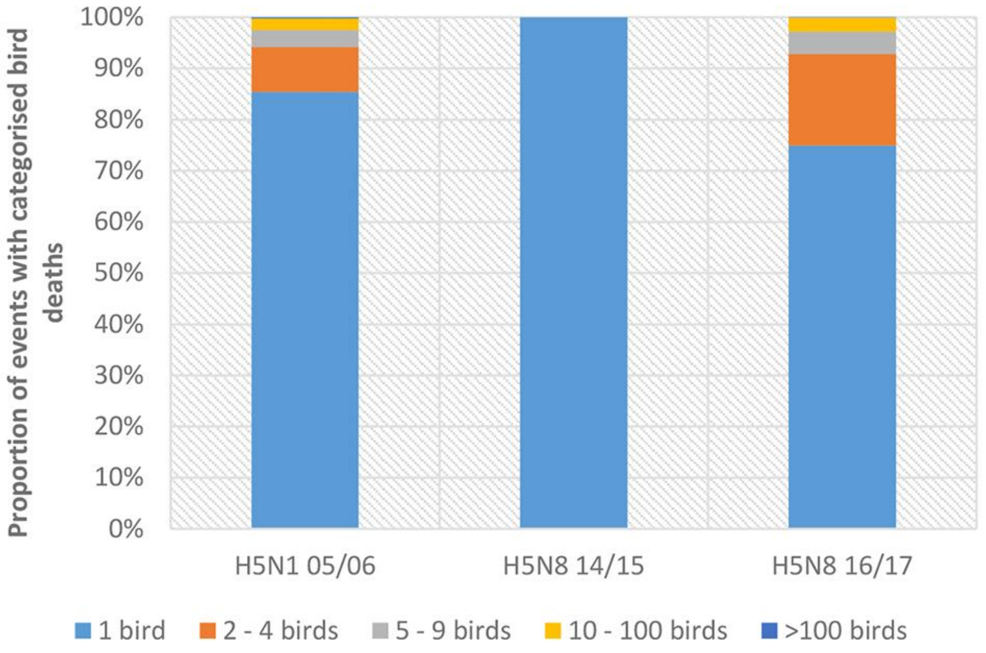

Technical Appendix Figure 2. Distribution of wild birds' incidents per category of number of deaths reported in the 3 epizootics.

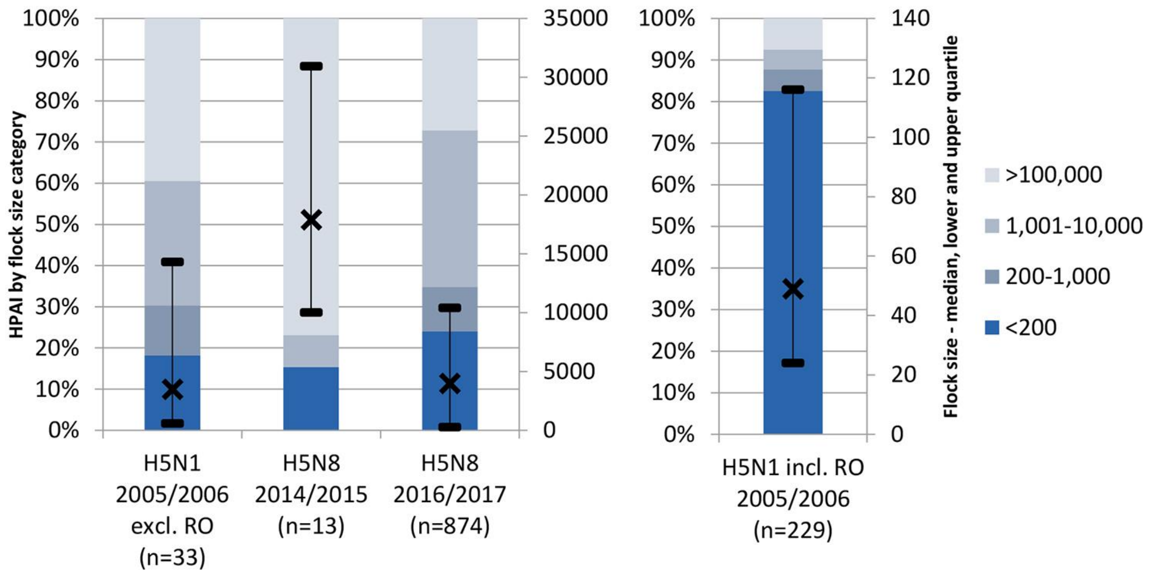

Technical Appendix Figure 3. Distribution of poultry flock sizes with a detection of H5 highly pathogenic avian influenza in 3 European epizootics. Crosses represent the median flock size and the interquartile intervals. RO, Romania.

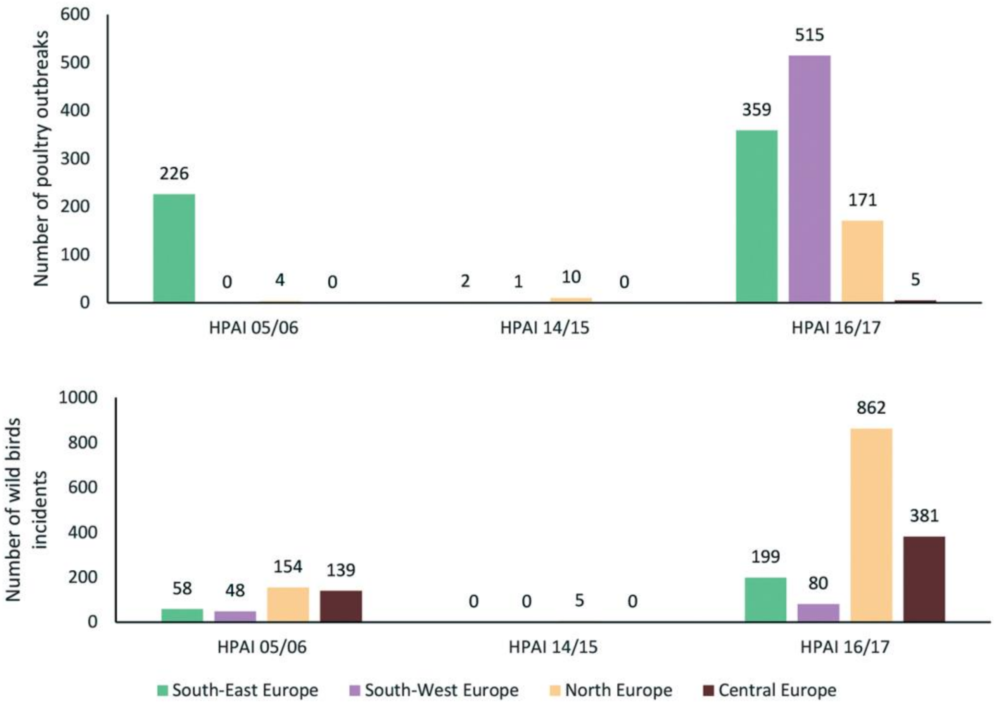

Technical Appendix Figure 4. Number of poultry outbreaks (top) and wild bird incidents (bottom) reported in four European regions.

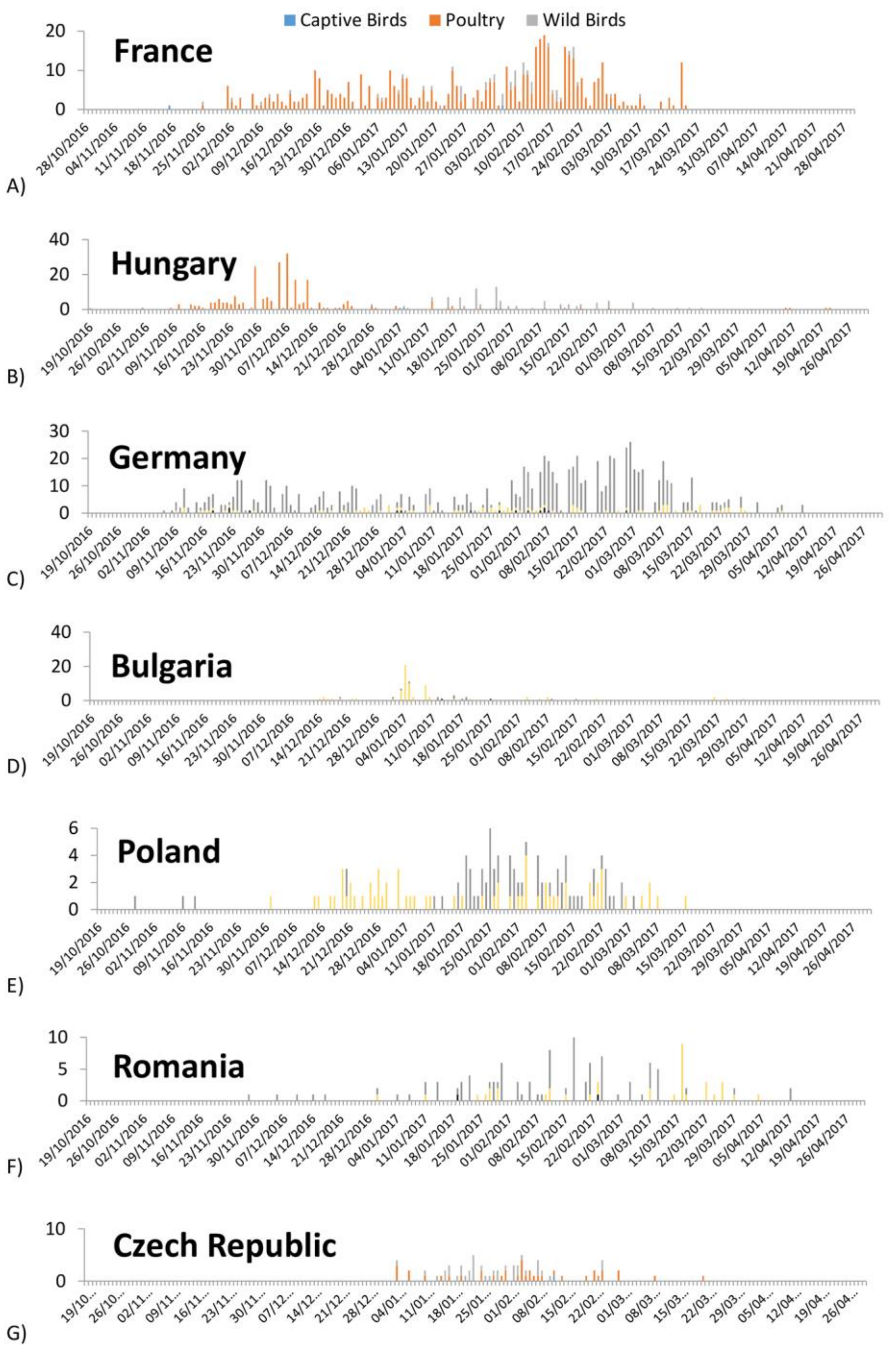

Technical Appendix Figure 5. Epidemic curve of the 2016–17 HPAI H5N8 epizootic in Europe (until April 30, 2017): A) France; B) Hungary; C) Germany; D) Bulgaria; E) Poland; F) Romania; G) Czech Republic.

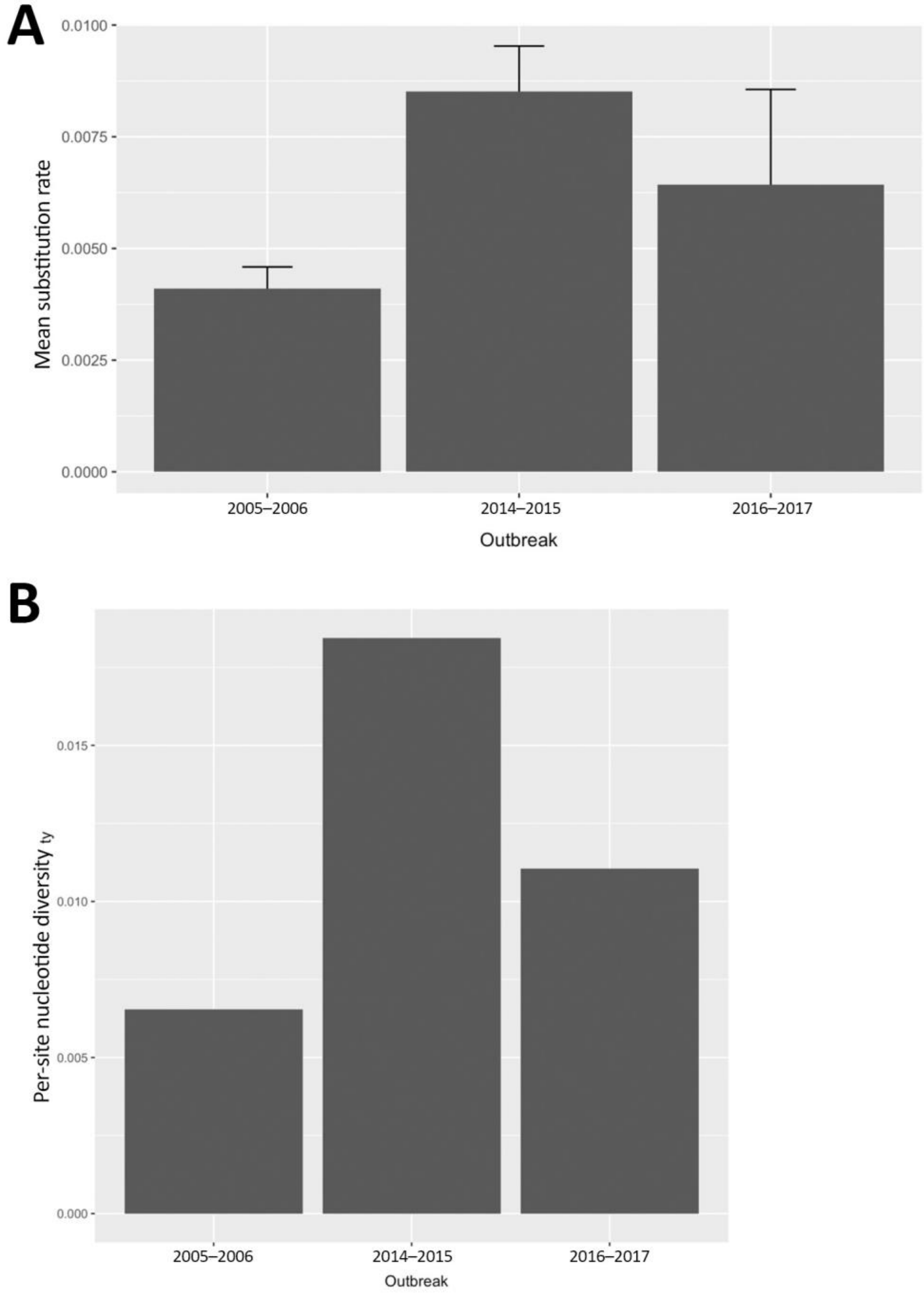

Technical Appendix Figure 6. A) Comparison of mean substitution rates (measured per site per year, with standard deviation) of H5 viruses in 2005–06, 2014–15, and 2016–17 outbreaks. B) Comparison of per-site nucleotide diversity, defined as average number of nucleotide differences per site between 2 DNA sequences in all possible pairs, in the sample population for viral population HA for each outbreak.

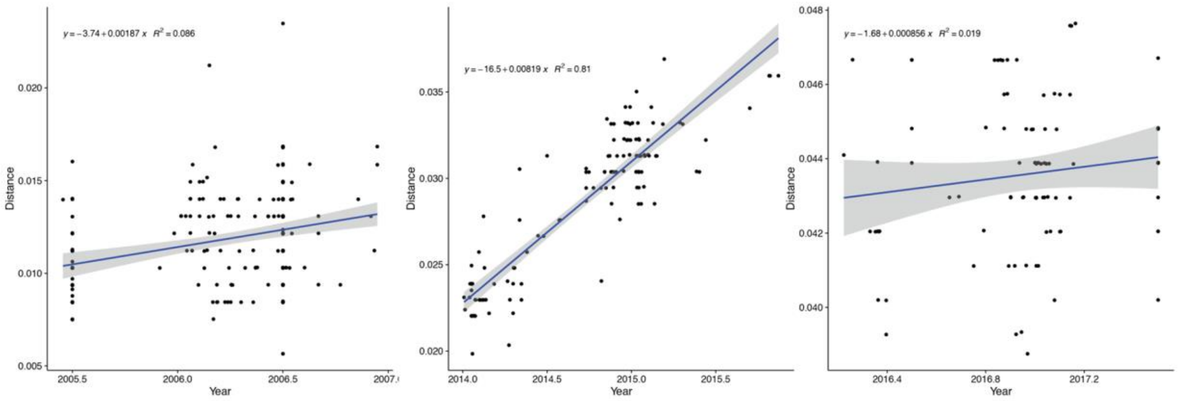

Technical Appendix Figure 7. Root to tip regression for ML trees generated from 2005–06, 2014–15, and 2016–17 outbreak viruses.

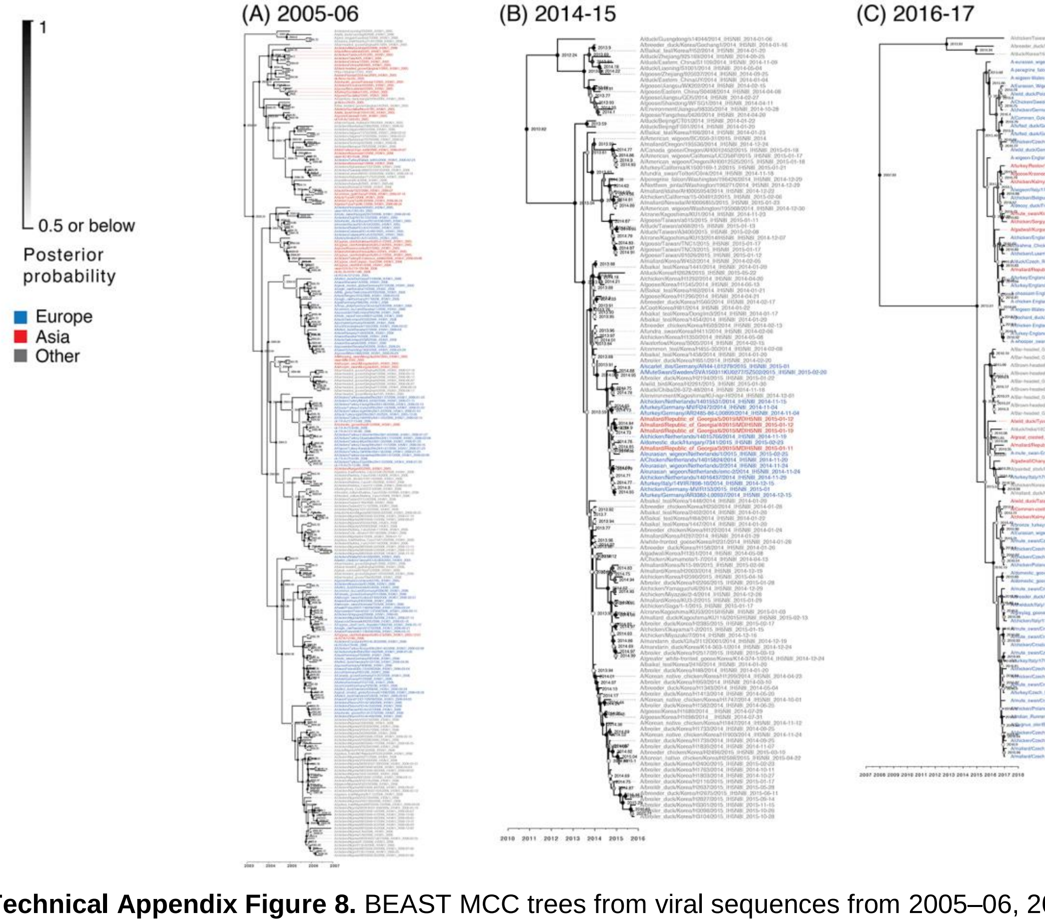

Technical Appendix Figure 8. BEAST MCC trees from viral sequences from 2005–06, 2014–15, and 2016–17 outbreaks of highly pathogenic avian influenza in Europe. Circles represent node support values, filled according the posterior probability values ranging from 0–1. Clades with European isolates are highlighted in gray. In the 2016–17 tree, black arrows represent unresolved European sequences.

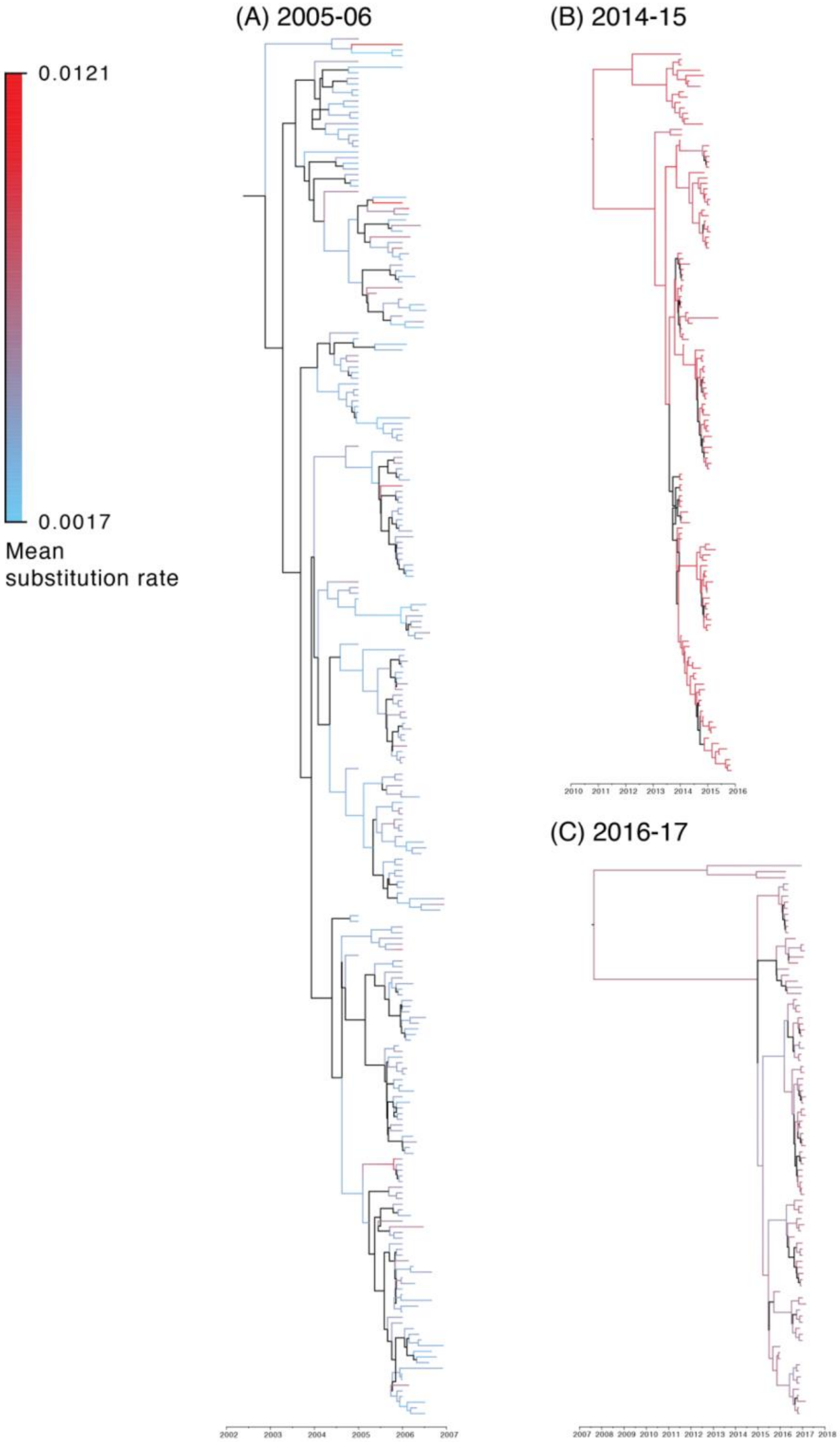

Technical Appendix Figure 9. BEAST MCC trees from downsampled viral sequences from 2005–06, 2014–15, and 2016–17 outbreaks of highly pathogenic avian influenza in Europe. Minimum and maximum rates are normalized across the 3 trees to enable comparison of rates.
